# Prevalence of urinary colonization by extended spectrum-beta-lactamase *Enterobacteriaceae* among catheterised inpatients in Italian long term care facilities

**DOI:** 10.1186/1471-2334-13-124

**Published:** 2013-03-06

**Authors:** Luca Arnoldo, Roberta Migliavacca, Laura Regattin, Annibale Raglio, Laura Pagani, Elisabetta Nucleo, Melissa Spalla, Francesca Vailati, Antonella Agodi, Adriana Mosca, Carla Zotti, Stefano Tardivo, Ines Bianco, Adele Rulli, Paola Gualdi, Pietro Panetta, Carlo Pasini, Mino Pedroni, Silvio Brusaferro

**Affiliations:** 1 Department of Medical and Biological Sciences, University of Udine, Udine, Italy; 2 Department of Clinical Surgical Diagnostic and Paediatric Sciences, Sect. of Microbiology, University of Pavia, Pavia, Italy; 3 Azienda per i Servizi Sanitari 3 - Alto Friuli, Udine, Italy; 4 Microbiology and Virology, AO Ospedali Riuniti di Bergamo, Bergamo, Italy; 5 University of Catania, Catania, Italy; 6 University of Bari, Bari, Italy; 7 University of Torino, Turin, Italy; 8 University of Verona, Verona, Italy; 9 Azienda Sanitaria Locale 2 Lanciano Vasto Chieti, Chieti, Italy; 10 Azienda provinciale per i servizi sanitari – Provincia autonoma di Trento, Trento, Italy; 11 Azienda Sanitaria Locale Taranto, Taranto, Italy; 12 Azienda Ospedaliera di Desenzano del Garda, Desenzano del Garda, Italy

## Abstract

**Background:**

Long Term Care Facilities (LTCFs) play a key role in guaranteeing care to patients in developed countries. Many patients, mostly elderly, access LTCFs at some time in their lives, and their healthcare pathways often require them to move back and forth between hospital and outpatient settings. These patterns bring about new challenges regarding infection control, especially healthcare associated infections.

**Methods:**

A point prevalence study was conducted in 23 Italian LTCFs, to identify colonization in patients with urinary catheter (>24 hours). Species identification, susceptibility tests and extended spectrum beta lactamase (ESBL) production screenings were performed using Vitek 2 System. Enterobacteria identified by Vitek 2 System as ESBL-producers or suspected AmpC hyperproducers on the basis of cephamycin resistance, were sent to a research laboratory where they underwent a double-disk synergy test. Finally, ESBL-producers were screened for *bla* resistance genes by PCR assay.

**Results:**

211 patients with catheter were screened, 185 out of 211 patients showed positive samples for the presence of *Enterobacteriaceae*, 114 of these 185 patients were colonized by extended spectrum cephalosporins resistant microorganisms. We identified a total of 257 Gram negative pathogens, of which 51.8% (133/257) were extended spectrum cephalosporins resistant. 7 out of 133 cephamycin not susceptible strains proved to be AmpC-type beta-lactamases and 125/133 ESBL-producers; 1 was not further characterized. 43 out of 257 (16.7%) *E. coli*, 37/257 (14.4%) *P. mirabilis*, 20/257 (7.8%), *P. stuartii*, 14/257 (5.4%) *M. morganii*, 7/257 (2.7%), *K. pneumoniae*, 4/257 (1.6%) *C. koseri* proved to be overall ESBL-producers by double-disk synergy test. Third and fourth generation cephalosporin resistant *P. mirabilis*, *P. stuartii* and *M. morganii* strains mainly harboured a *bla*TEM gene (95.9%), while 89.1% of *E. coli* were positive for the *bla*CTX-M determinant by PCR and sequencing. Patients with decubitus had a higher risk of colonization by at least one resistant isolate (p < 0.01). Samples of patients undergoing antibiotic therapy and patients with decubitus showed a higher risk (p < 0.05) of colonization by beta-lactam resistant microorganisms.

**Conclusions:**

These data confirm the presence of high percentages of ESBL-positive Enterobacteria in Italian LTCFs and the predominance of CTX-M type ESBL in *E. coli*. The alarming presence of ESBL-producing *Enterobacteriaceae* in Italian LTCFs can seriously compromise the effectiveness of antibiotic therapy.acilities (LTCFs), Antimicrobial resistance.

## Background

Long Term Care Facilities (LTCFs) play a key role in guaranteeing care to patients in developed countries. Many patients, mostly elderly, access LTCFs at some time in their lives, and their healthcare pathways often require them to move back and forth between hospital and outpatient settings [1].

These patterns bring about new challenges regarding infection control, especially healthcare associated infections (HAI). This is also true considering that LTCFs differ from acute intensive care settings in clinical approaches, models, organization of care, and quantity and quality of human resources. Nevertheless, they usually make a limited use of diagnostic and microbiological tests [2] and therefore, laboratory surveillance suffers from the lack of this kind of approach in prescribing specific tests.

Antibiotic resistance is consistently present in these settings, and this has a critical impact also because patients frequently move back and forth within and between other healthcare organizations [3-5].

A better knowledge of the epidemiology of antibiotic resistance patterns in LTCFs is necessary, because the spread of Extended Spectrum Beta-Lactamases (ESBL) and recently of Beta-Lactamases (BL) with carbapenemasic activity (e.g. *K. pneumoniae* KPC producer) [6], has emerged as a major source of antibiotic resistance in gram-negative pathogens, and this is one of the most important causes of antibiotic therapy failure [7-9]. The emergence of ESBL-producers has important clinical and therapeutic implications because these microorganisms are responsible for both hospital and community acquired infections [10,11].

Point prevalence surveys are useful tools to conduct an initial study of the spread of resistant microorganisms in a population at risk such as the elderly, to understand the burden of the problem, and to define a benchmark of resistance genes to control their development.

ESBL acquisition is associated with the following risk factors: presence of decubitus, presence of a gastrostomy tube, poor functional condition, prior use of ciprofloxacin, prior use of trimethoprim-sulfamethoxazole [12,13]. These clinical risk factors refer to a specific geriatric population that is largely predominant in LTCFs.

Although ESBL-producers are a major concern in the clinical field and their presence in different Italian settings has been reported, nationwide data on their prevalence in LTCF residents are not available [14].

The aim of this study is to detect the circulation of BL-producing *Enterobacteriaceae,* particularly ESBL, in Italian LTCFs.

## Methods

We conducted a point prevalence study in 23 Italian LTCFs at the end of 2006, looking for colonization in patients with urinary tract catheters (>24 hours) at the time of the study. In each LTCF, the point prevalence survey began and was completed within an index day. Eight contact centres (CCs) were set up in different parts of the country as reference centres for the local LTCFs (involved on a voluntary basis); each CC had to guarantee:

•a minimum of 9 patients

•filled-in patient data forms;

•presence of laboratory with VITEK 2 technology;

•possibility of the laboratory to isolate strain and freeze it for later use.

Patients joined the study on a voluntary basis after signing an informed consent statement. Patient data were used anonymously. At the time of the study, there was no legal obligation (DM 15/07/1997 and DM 12/05/2006) to obtain the approval of an Ethic Committee prior to conducting an observational (non experimental) studies.

Urine samples, collected by nursing staff directly from the catheter valve, were sent to CC laboratories for culture and species identification of the Enterobacteria present in all samples (by inoculation of 0,001 mL on McConkey agar, incubation at 35°C for 24 h). Species identification, susceptibility tests and ESBL-production screenings were carried out using 21341-GN and 22088-AST-N041 cards, Vitek 2 System (Bio-Mèrieux). Enterobacteria identified as ESBL-producers by Vitek 2 System or as suspected AmpC hyperproducers due to their cephamicin resistance, were stocked and sent to the research laboratory of the University of Pavia, where the microorganisms were subjected to both the double-disk synergy test using piperacillin-tazobactam (TZP) and cefotaxime (CTX), cefepime (FEP), ceftazidime (CAZ) and aztreonam (ATM), and to the combo confirmatory test according to CLSI recommendations [15].

All ESBL-producers were screened for *bla*_SHV_, *bla*_TEM_, *bla*_CTX-M_, and *bla*_OXA_ resistance genes by PCR assay, using primers as summarized in Table 1. Control organisms included strains of *E. coli* containing either *bla*_TEM-1_, *bla*_SHV-2_, *bla*_OXA-4_, *bl*a_CTX-M-15_ or *bla*_CTX-M-9_ genes.

**Table 1:** Primers used for the detection of the presence of different beta lactamases genes by PCR

**Amplicon**	**Primer sequence (5**^′^**to 3**^′^**)**	**Size (bp)**
bla_SHV_	GCCCGGGTTATTCTTATTTGTCGC	900 [16]
	TCTTTCCGATGCCGCCGCCAGTCA	
bla_TEM_	ATAAAATTCTTGAAGACGAA	960 [17]
	ATATGAGTAAGCTTGGTCTGACAG	
bla_CTX-M_	ATGTGCAGYACCAGTAARGT	593 [18]
	TGGGTRAARTARGTSACCAGA	
bla_CTX-M-3/15/22_	GTTACAATGTGTGAGAAGCAG	1000 [18]
	AACGGAATGAGTTTCCCCATT	
bla_CTX-M gr 9_	ATGGTGACAAAGAGAGAGTGCA	835 [19]
	CCCTTCGGCGATGATTCTC	
bla_OXA-1_	ACA CAA TAC ATA TCA ACT TCG C	813 [20]
	AGT GTG TTT AGA ATG GTG ATC	

Given the previously reported description of CMY-16 in *P. mirabilis* from northern Italy [21], *P. mirabilis* clinical isolates were also screened for the presence of the *bla*_CMY-16_ gene, using specific primers CMY-Exp_Fw GCTCTAGACATATGATGAAAAAATCGTTATGCTG CMY-Exp_Rev CGGGATCCTTATTGCAGCTTTTCAAGAATG (1165 bp amplicon size).

A multiplex-PCR protocol was used to identify family-specific AmpC genes responsible for AmpC BL expression in organisms with or without a chromosomal AmpC BL gene [22]. Analytical isoelectric focusing (IEF) of crude cell extracts, visualization of BL bands by nitrocefin (gels were electrophoresed at 11 to 14 W for 90 min and BL revealed with 0.5 mM Nitrocefin) and detection of their activity by a substrate overlaying procedure, were performed as described.

Known producers of different BL (i.e., TEM-1, TEM-2, TEM-7, TEM-8, TEM-9, TEM-12, SHV-1, SHV-2 and SHV-5) were used as controls.

PCR amplicons of *bla*_TEM,_*bla*_SHV,_*bla*_OXA,_*bla*_CTX-M_ genes were purified using the kit Quantum Prep PCR Kleen Spin Columns (BioRad) and subjected to direct sequencing. PCR products were sequenced on both strands with Applied Biosystems sequencers.

The nucleotide sequences were analysed according to the BLAST program.

For each patient, a standardized form was filled in; it included variables related to reported risk factors for HAI in LTCFs [23]:

•patient data (gender, age, residence prior to admission to LTCF, surgery in the previous 30 days, hospital admission in the previous six months and discharge ward);

•co-morbidities (e.g. diabetes, cancer, renal failure);

•use of devices (central venous catheter CVC, percutaneous endoscopic gastrostomy PEG);

•clinical data (presence of decubitus, chemotherapy or therapy with steroids in the previous 30 days, leucocytosis, on-going antibiotic therapy).

Both microbiological and epidemiological data were collected in a database and statistical analysis was performed using SPSS 12. Variables were compared using chi square test and logistic regression. We accepted p < 0.05 as significant.

## Results and discussion

**Figure 1:**
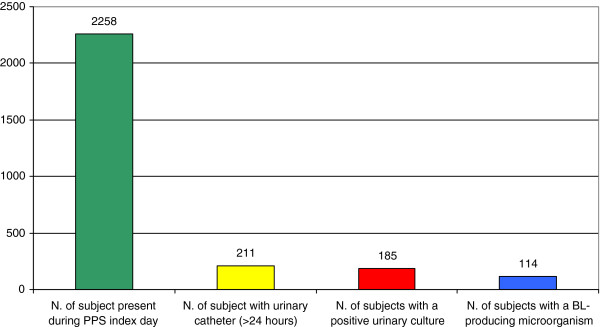
Number of individuals present during PPS, with a urinary catheter, with a positive culture and with a BL-producing microorganism.

We enrolled all patients with urinary catheter (>24 hours), i.e. 211 patients out of the 2258 who were present in the 23 LTCFs (Figure 1); 149 patients (70.6%) were in northern Italy, 9 (4.3%) in central Italy and 53 (25.1%) in southern Italy.

Females were 61.6% (130/211), mean age was 83.3 (male 80.4, female 85.2). 68 patients (32.2%) had been in the LTCF for over 6 months. 26 patients (12.3%) had been hospitalized in the 30 days prior to entering the LTCF. These patients were discharged from the following wards: internal medicine 15 (57.7%), specialist surgery 8 (30.8%), general surgery 2 (7.7%), onco-hematology 1 (3.8%). 29 patients out of 211 (13.7%) had ongoing antibiotic therapy, and 5 of these were administered a multidrug antibiotic therapy. Length of catheterization was ≤1 month for 130 out of 191 patients (68.1%) and >1 month for 61 out of 191 (31.9%).

Thirty-four antibiotics were reported altogether: 44.2% (15/34) were beta-lactams (7 penicillins, 7 cephalosporins and 1 carbapenem), 29.4% (10/34) quinolons, 8.8% (3/34) tetracyclines, 8.8% (3/34) macrolides, 5.9% (2/34) glycopeptides and 2.9% (1/34) trimethoprim-sulfamethoxazole.

56.9% (120/211) of the patients had no reported co-morbidities, 24.2% (51/211) had diabetes mellitus, 14.2% (30/211) had renal failure and 12.8% (27/211) had cancer.

A total of 257 microorganisms were isolated from 185 patients; 62.2% (119/185) of urinary samples showed the presence of one single microorganism, 32.4% (60/185) of two bacteria and 3.2% (6/185) of 3 different species.

Phenotypical and molecular tests confirmed BL-positive results for 133/257 strains (51.8%): 125 strains were ESBL-producers and 7 AmpC hyper producers; one strain was not screened. Double-disk synergy and combo confirmatory tests yielded positive results for all ESBL-producers. AmpC producers were resistant to expanded-spectrum cephalosporins (with the exception of FEP), ATM and cephamycins, while susceptibility to carbapenems was not affected.

114 patients had at least one resistant BL-producer strain.

Analytical IEF analysis revealed the presence of at least one BL band with pIs values ranging from 5.4 to >8.4 in all the above-mentioned isolates (data not shown).

The total number of BL bands and the ability of each enzyme to hydrolyze extended-spectrum cephalosporins (CTX, CAZ, ATM, FEP) and/or FOX, suggested the production - in different strains - of ESBL or of AmpC-type BL (CBL).

In 4 *P. mirabilis* strains, PCR and sequencing revealed and confirmed the presence of an identical allele in all isolates, which encoded the CMY-16 variant.

The ESBL- positive isolates included 43 *E. coli*, 37 *P. mirabilis*, 20 *P. vulgaris*, 14 *M. morganii*, 7 *K. pneumoniae* and 4 *C. koseri*.

*E. coli*, *P. mirabilis* and *P. stuartii* altogether proved to be the most commonly isolated microorganisms, and *P. mirabilis, P. stuartii* and *M. morganii* showed a higher presence of ESBL.

**Figure 2:**
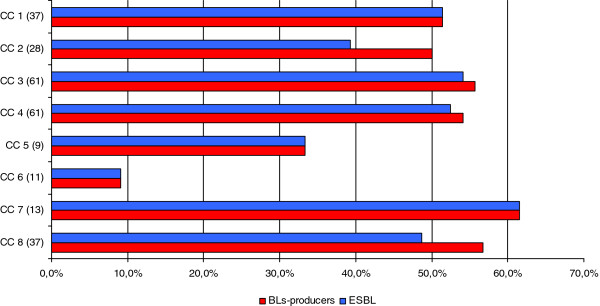
Distribution of BL and ESBL strains stratify by eight Contact Centres (in parentheses the number of microorganisms isolated for each CC).

Table 2 summarizes the total number of different species collected in the study, indicating the ones that were positive to ESBL confirmatory tests. There was no statistical significance (chi square test) in prevalence resistance between northern and central-southern Italy LTCFs; Figure 2 shows the percentage of BL-producing strains and ESBL in the different CCs.

**Table 2:** Number and percentage of BL and ESBL producers

**Isolated microorganisms**			**Beta lactamases producers**		**ESBL**	
**Specie (n.)**	**North**	**Centre-south**	**North**	**Centre-south**	**North**	**Centre-south**
	**n.**	**n.**	**n. (%)**	**n. (%)**	**n. (%)**	**n. (%)**
*Escherichia coli (110)*	78	32	37 (47.4)	9 (28.1)	34 (43.6)	9 (28.1)
*Proteus mirabilis (51)*	30	21	20 (66.7)	18 (85.7)	20 (66.7)	17 (81.0)
*Providencia stuartii (31)*	25	6	17 (68.0)	5 (83.3)	16 (64.0)	4 (66.7)
*Klebsiella pneumoniae (26)*	20	6	7 (35.0)	0 (−)	7 (35.0)	0 (−)
*Morganella morganii (23)*	20	3	14 (70.0)	0 (−)	14 (70.0)	0 (−)
*Citrobacter koseri (7)*	7	0	4 (57.1)	0 (−)	4 (57.1)	0 (−)
*Enterobacter cloacae (4)*	2	2	0 (0.0)	1 (50.0)	0 (−)	0 (−)
*Serratia marcescens (3)*	3	0	1 (33.3))	0 (−)	0 (−)	0 (−)
*Proteus vulgaris (1)*	1	0	0 (0.0)	0 (−)	0 (−)	0 (−)
*Citrobacter freundii (1)*	1	0	0 (0.0)	0 (−)	0 (−)	0 (−)
Total (257)	187	70	100 (53.5)	33 (47.1)	95 (50.8)	30 (42.9)

*E. coli* was the most common microorganism detected both in northern areas (CC1, CC2, CC3, CC4) and in central-southern areas (CC5, CC6, CC7, CC8), but while in northern LTCFs it was also the most frequent BL-producer, the most commonly resistant strain in central-southern LTCFs was *P. mirabilis*. The latter microorganism also showed geographical differences that were statistically significant (OR 1.7 CI 95% 1.06-2.72 p < 0.01), in the expression of TEM-types gene, with TEM-92 present in 90.0% (18/20) of the strains in northern Italy and 52.9% (9/17) of TEM-92 in centre-southern Italy where the remaining ones were TEM-4 or TEM-24; Table 3 summarizes the PCR/sequencing results for each contact centre.

**Table 3:** PCRs/sequencing results for each contact centre

**Contact centres (n. of microorganisms)**	**Strain**	**N° of strains BL and ESBL patterns**
CC 1	*E. coli (9)*	2 (CTX-M-1. TEM-1).
		3 (CTX-M-19. TEM-1).
		2 (CTX-M-15. OXA-30. TEM-1).
		1 (CTX-M-32). 1 (SHV-2.TEM-1)
	*K. pneumoniae (1)*	1 (SHV-1 TEM-151)
	*P. mirabilis (2)*	1 (TEM-4). 1 (TEM-92 + CMY-16)
	*M. morganii (1)*	1 (TEM-92)
	*P. stuartii (6)*	5 (TEM like). 1 (TEM-60)
CC 2	*E.coli (5)*	1 (CTX-M-3. TEM-1).
		1 (CTX-M-15. OXA-30). 1 (SHV-5.TEM-1).
		2 (AmpC overexpress)
	*K. pneumoniae (1)*	1 (CTX-M-19 o 54 SHV-1)
	*P.mirabilis (2)*	1 (TEM-92 TEM-2).
		1 (TEM-92)
	*M .morganii (5)*	5 (TEM-92)
	*P. stuartii (1)*	1 (AmpC)
CC3	*E. coli (5)*	2 (CTX-M-15. OXA-1-like).
		1 (CTX-M-15. OXA-30. TEM-1).
		1 (CTX-M-3. OXA-1-like. TEM-1). 1 (CTX-M-32)
	*K. pneumoniae (3)*	2 (CTX-M-3. SHV-1). 1 (CTX-M-15. SHV-1)
	*P. mirabilis (10)*	8 (TEM-92). 2 (TEM-92 + CMY-16)
	*M. morganii (6)*	6 (TEM-92)
	*P. stuartii (5)*	4 (TEM-like). 1 (TEM-60)
	*C. koseri (4)*	4 (TEM-134. TEM-1)
	*S. marcescens (1)*	1 (AmpC)
CC 4	*E. coli (18)*	4 (CTX-M-15. OXA-30).
		1 (CTX-M-15. OXA-30. TEM-1).
		6 (CTX-M-15. TEM-1).
		1 (CTX-M-32. TEM-1).
		5 (CTX-M-15. OXA-1-like. TEM-1). 1 (AmpC overexpress)
	*K. pneumoniae (2)*	1 (CTX-M-15. SHV-1). 1 (CTX-M-3. SHV-1)
	*P. mirabilis (6)*	5 (TEM-92). 1 (TEM-4)
	*M. morganii (2)*	2 (TEM-92)
	*P. stuartii (5)*	3 (TEM-like). 2 (TEM-60)
CC 5	*E. coli (2)*	2 (CTX-M-15. OXA-30. TEM-1)
	*P. mirabilis (1)*	1 (TEM-92 + CMY-16)
CC 6	*P. mirabilis (1)*	1 (TEM-92)
CC 7	*E. coli (2)*	1 (CTX-M-19. TEM-1).
		1 (CTX-M-15. OXA-1-like. TEM-1)
	*P. mirabilis (2)*	1 (TEM-4).
		1 (TEM-24)
	*P. stuartii (4)*	4 (TEM-like)
CC 8	*E. coli (5)*	2 (CTX-M-15. OXA-30. TEM-1).
		3 (CTX-M-14. TEM-1)
	*P. mirabilis (14)*	6 (TEM-4). 7 (TEM-92). 1 (no screened)
	*P. stuartii (1)*	1 (AmpC)
	*E. cloacae (1)*	1 (AmpC chromosomal)

Table 4 shows the results of bivariate analysis for patients with the presence of BL-producing microorganisms based on reported characteristics. Also length of catheterization shows no statistically significant differences. In the logistic regression model (Table 4), we included all the variables, and patients with decubitus emerged as those with a statistically significant higher risk of detecting at least one resistant microorganism (OR 3.22 CI 95% 1.57-6.60 p < 0.01). Furthermore, we conducted the same analysis for microorganisms (257) and found that 50 out of 80 (62.5%) microorganisms that came from patients with decubitus were resistant (chi square test OR 1.89 CI 95% 1.10-3.24 and logistic regression OR 2.13 CI 95% 1.14-4.00 p < 0.05); this strengthens the thesis that patients with decubitus are at a higher risk of selecting resistant strains.

**Table 4:** General characteristics of studied population and results of bivarite analysis and logistic regression model (211)

**Population characteristics**	**N. (%) of residents with risk factors**		**Bivariate analysis**		**Logistic regression model**		
	**ESBL/AmpC (−) (n. 97)**	**ESBL/AmpC (+) (n. 114)**					
	**n.**	**n.**	**OR**	**CI 95%**	**OR**	**CI 95%**	**p°**
Stay longer than 6 months	38	30	0.56	0.31-0.99	0.57	0.29-1.10	ns
Hospital admission 30 days before	17	9	0.40	0.17-0.95	0.47	0.15-1.44	ns
Surgery 30 days before	5	2	0.33	0.62-1.73	0.36	0.05-2.75	ns
Antibiotic therapy	13	16	1.06	0.48-2.32	1.27	0.52-3.08	ns
Central Venous Catheter	3	1	0.28	0.3-2.71	0.22	0.19-2.46	ns
Pecutaneous Endoscopic Gastrotomy	8	15	1.69	0.68-4.17	1.28	0.46-3.57	ns
Decubitus	21	43	2.19	1.19-4.05	3.22	1.57-6.60	p <0.01
Co-morbodities*	44	47	0.85	0.49-1.46	0.94	0.52-1.69	ns

*include cancer, diabetes and renal failure.

°ns = no statistically significant.

Moreover, we found that 18 out of 24 (75%) strains that came from patients undergoing antibiotic therapy showed a high risk of being resistant (chi square test OR 3.08 CI 95% 1.18-8.03 and logistic regression OR 4.70 CI 95% 1.56-14.22 p < 0.01).

This study investigated the circulation of BL-producing *Enterobacteriaceae* in Italian LTCFs. It also analyzed the distribution of the beta-lactam resistant genes throughout the entire national territory and it produced a first nationwide picture of the issue.

We found an overall remarkably high presence of BL-producers 51.8% (133/257), especially of ESBL-producers 48.6% (125/257) in Italian LTCFs, even with variations among centres. We found a high percentage of ESBL circulation particularly for *P. mirabilis* 72.5% (37/51) [24], *P. stuartii* 64.5% (20/31) and *M. morganii* 60.9% (14/23); these data confirm the increase of ESBL-producers in LTCFs reported in other studies [25,26]. Moreover they confirm that CTX-M-type ESBL are the most widespread in *E. coli*, particularly CTX-M-15 [27]; this gene is also the one that was most frequently isolated in acute intensive care hospitals in Italy in other studies [14,28].

Probably, using the CLSI confirmatory test allows to correctly identify the typical ESBL, but it could increase the risk of underestimating the presence of plasmid AmpC-producers. Some authors have already published papers on the spread of CMY-16 *P. mirabilis*, which mainly occurs in northern Italy [21,29].

The aim of the study was to obtain a picture of ESBL circulation in LTCFs. Moreover, in order to better control the existing case mix variability among LTCFs, we selected only patients with a permanent urinary catheter. Nevertheless, the population characteristics are similar to those reported in literature for these patients [5]: prolonged length of stay, high prevalence of co-morbidities, high mean age.

When we explored possible correlations with population variables, we were able to confirm that there is a significant correlation between the presence of decubitus and the presence of at least one beta-lactam resistant microorganism, as was previously reported.

We also found that patients with an ongoing antibiotic therapy had a higher number of expanded-spectrum cephalosporins resistant microorganisms among the isolates, compared to patients without antibiotic therapy. This confirms what is reported in other studies [30].

We selected patients characteristics and clinical risk factors that were compatible with a point prevalence study, but this could have limited the capacity to make clear the role of other factors such as previous use of antibiotic in the population. Relationships with decubitus ulcers must be further explore to be not confounded by other clinical factors that are not included in our model.

Due to the sample dimension and to the patient selection criteria (we limited the study only to patients with urinary catheter), this study certainly cannot be considered as being completely representative of the Italian BL situation, but it does provide a first picture of the problem in the crucial healthcare sector of LTCFs; moreover, it shows the need for urgent investments both in monitoring and controlling antibiotic resistance in LTCFs.

## Conclusions

This study confirmed the wide circulation of BL-producing *Enterobacteriaceae* in LTCFs, and particularly the increase of ESBL-producers (CTX-M). This is important to improve effective and appropriate therapeutic choices in LTCFs, where there are fewer opportunities of using microbiological tests and patient conditions (elderly people with many co-morbidities) may easily lead to therapeutic failure. Moreover, since these patients need to access other healthcare settings quite frequently, this increases the risk of spreading the ESBL among healthcare organizations. Our data confirm a different circulation of expanded-spectrum cephalosporins resistant microorganisms in Italy. This indicates that local monitoring of circulating ESBL-types is relevant and important, since it can provide information about resistance evolution which, in turn, can help make appropriate therapeutic decisions. In healthcare systems, managing LTC is becoming more relevant, and monitoring antibiotic resistances in these settings (including carbapenems, often one of the few remaining therapeutic options) is essential in order to guarantee an appropriate use of antibiotics and high quality of patient care.

## Abbreviations

LTCFs: Long term care facilities; ESBL: Extended spectrum beta-lactamases; BL: Beta-lactamases; HAIs: Healthcare associated infections; LTC: Long term care; CC: Contact centre; PEG: Percutaneous endoscopic gastrostomy; CVC: Central venous catheter; PCR: Polymerase chain reaction

## Competing interests

The authors declare that they have no competing interests.

## Authors’ contributions

BS and RL designed the study. AL performed the statistical analysis, analysed and interpreted data and drafted the manuscript. RL collected data and drafted the manuscript. MS and EN collected all bacterial strains and related data and performed antibiotic susceptibility tests. AA, BI, RA, GP, TS, MA, ZC, PP, PC and PM collected data from LTCFs and coordinated CC laboratory work. MS, EN and RM carried out experimental screening work aimed at BL/ESBL detection; RM and EN performed the molecular characterization of the BL/ESBL. RM drafted the manuscript. LP supervised both the BL/ESBL screening work and the genes characterization activity. RA coordinated microbiological work and drafted the manuscript. RA and VF made phenotypic confirmation. BS coordinated epidemiological work, analysed and interpreted data and drafted the manuscript. The manuscript was revised and approved by all authors.

## Request for consent statement

All patients participated in the study on a voluntary basis after signing an informed consent. Patient data were used anonymously. At the time of the study, there was no legal obligation to obtain the approval of an Ethic Committee (DM 15/07/1997 and DM 12/05/2006) prior to conducting an observational (non experimental) studies. At the time of the study the Ethic Committee was mandatory only for experimental studies.

## Pre-publication history

The pre-publication history for this paper can be accessed here:

http://www.biomedcentral.com/1471-2334/13/124/prepub
